# Chronic Disease and Its Risk Factors Among Refugees and Asylees in Massachusetts, 2001-2005

**Published:** 2010-04-15

**Authors:** Nameeta M. Dookeran, Tracy Battaglia, Jennifer Cochran, Paul L. Geltman

**Affiliations:** Brigham and Women’s Hospital, Boston, Massachusetts; Boston University School of Medicine, Boston, Massachusetts; Massachusetts Department of Public Health, Boston, Massachusetts; Refugee and Immigrant Health Program, Massachusetts Department of Public Health, Department of Pediatrics. Dr Geltman is also affiliated with Boston University School of Medicine.

## Abstract

**Introduction:**

Better understanding of the health problems of refugees and people who are granted political asylum (asylees) in the United States may facilitate successful resettlement. We examined the prevalence of risk factors for and diagnoses of chronic disease among these groups in Massachusetts.

**Methods:**

We retrospectively analyzed health screening data from 4,239 adult refugees and asylees who arrived in Massachusetts from January 1, 2001, through December 31, 2005. We determined prevalence of obesity/overweight, hypertension, coronary artery disease (CAD), diabetes, and anemia. Analyses included multivariate logistic regression to determine associations between CAD and diabetes with region of origin.

**Results:**

Almost half of our sample (46.8%) was obese/overweight, and 22.6% had hypertension. CAD, diabetes, and anemia were documented in 3.7%, 3.1%, and 12.8%, respectively. People from the Europe and Central Asia region were more likely than those from other regions to have CAD (odds ratio, 5.55; 95% confidence interval, 2.95-10.47).

**Conclusions:**

The prevalence of obesity/overweight and hypertension was high among refugees and asylees, but the prevalence of documented CAD and diabetes was low. We noted significant regional variations in prevalence of risk factors and chronic diseases. Future populations resettling in the United States should be linked to more resources to address their long-term health care needs and to receive culturally appropriate counseling on risk reduction.

## Introduction

The United States has a longstanding humanitarian commitment to the resettlement of refugees from overseas. Each year, the number of people granted refugee and political-asylum status in the United States fluctuates based on variations in the stability of other countries, the global political climate, and domestic resettlement targets. The largest number of refugee admissions in 2008 was to the United States (68% of the 80,800 resettled refugees worldwide), but Australia, Canada, and Sweden had higher per capita admission rates (52.4, 32.5, and 24.3 refugees per 100,000 residents, respectively) than the United States (19.8 refugees per 100,000 residents) (1-3). In the 3 years from 2006 through 2008, the United States approved an average of 24,750 claims for asylum per year; the 3 leading countries of nationality (China, Colombia, and Haiti) together constituted 36.4% to 44.0% of all approvals (4).

Refugees and asylees (people who are granted asylum) are people outside of their country of origin who are unable or unwilling to return to that country because they have experienced, or have a legitimate fear of, persecution on the grounds of race, religion, nationality, membership in a particular social group, or political affiliation (5). People who are granted refugee status and admission to the United States apply while overseas after having fled their home country or, for certain nationalities, while in-country. In contrast, people who seek political asylum do so either some time after entry into the United States or on arrival at a US port of entry. Historically, asylum applicants were in the United States for many years before being granted asylum because of delays in filing and processing asylum applications. Recently, this difference in time in the United States between refugees and asylees has lessened, in part because 1995 federal immigration legislation required potential asylees to file asylum applications within 1 year of arrival (6).

Because refugees and asylees differ in how long they have been in the United States, their countries of origin, and their socioeconomic circumstances, they likely have different health care needs. The Massachusetts Refugee Health Assessment Program (RHAP), a partnership between the Department of Public Health and contracted private, mostly federally qualified clinics, was established in 1995 to perform health screenings of refugees and other people who were eligible for refugee benefits. The latter include asylees, Cuban and Haitian entrants, certain Amerasians (mostly from Vietnam), and victims of human trafficking (7). Asylees were effectively denied access to RHAP services until 2000, when the starting date of time-limited eligibility for services was changed from the date of physical entry into the United States to the later date of asylum approval (8).

Domestic refugee health assessment programs, such as RHAP, have traditionally focused on identification and treatment of infectious diseases, although such programs also serve as a bridge to primary care. Few studies have focused on the screening of newly arrived refugees in the United States for chronic diseases, mental illness, or substance abuse, despite their relevance in these populations (9-14). Asylees may also be at risk of developing chronic diseases through acculturation while living as marginalized residents of low-income, urban neighborhoods in the United States before being granted asylum status.

The burden of chronic disease is high in many of the countries where refugees and asylees live before resettling in the United States. World Health Organization data show higher chronic-disease–related death rates in low- and middle-income countries compared with Canada or the United Kingdom (15). During the past 15 years, the largest group of refugees entering the United States has been from the nations that were formed from the former Soviet Union. Among this group, the Russian Federation in particular has seen growing mortality from preventable causes other than communicable disease, and cardiovascular disease is the leading cause of death (16). In the wake of the collapse of the Soviet Union, Russian life expectancy has declined as rates of nutritional deficiency and alcoholism have risen (17-19).

The changing demographics of both refugees and asylees entering the US health care system may result in greater health care needs for chronic, noninfectious diseases. However, programs designed to assess refugee health care needs are not generally structured to address chronic health problems. The objectives of this study were to determine the documented prevalence of risk factors for, and diagnoses of, chronic diseases among refugees and asylees who received RHAP health screening and to determine whether differences in prevalence of chronic disease and risk factors were associated with region of origin or visa category.

## Methods

We performed a retrospective cross-sectional study using RHAP data from health screenings of asylees and refugees. For the purposes of this article, the term "refugees" includes people newly arrived in the United States from overseas (ie, true refugees), derivative asylees (ie, people arriving from overseas to reunite with immediate family members previously granted asylum in the United States), and Cuban, Haitian, and Amerasian special entrants. Eligible participants were aged 18 years or older, had entered the United States from January 1, 2001, through December 31, 2005, and had completed the RHAP screening (7). The institutional review board of Boston University Medical Center approved and monitored the conduct of this study, and the Massachusetts Department of Public Health approved the public release of this data analysis.

In the RHAP electronic database, the Massachusetts Department of Public Health maintains clinical and public health data on asylees and refugees, derived from official government arrival notifications and RHAP reporting forms submitted by contracted health assessment clinical sites. Government arrival notifications are the source of basic demographic information (eg, patient age, sex, country of origin) and, in the case of refugees, medical diagnoses documented in reports from medical examinations performed overseas before arrival in the United States. RHAP reporting forms are the source of additional medical diagnoses and information obtained during refugee and asylee screening in the United States; they comprise a history and physical examination, immunizations, and a set of standard (eg, stool ova and parasites, complete blood counts, urinalyses) and optional tests based on individual health needs.

Risk factors for chronic disease included evidence of obesity (body mass index [BMI], ≥30 kg/m^2^) or being overweight (BMI, 25.0-29.9 kg/m^2^) and provider documentation of hypertension (including people with a single high blood pressure [systolic blood pressure ≥140 mm Hg] measurement) (20-22). Not all people with 1 elevated systolic blood pressure reading have true hypertension, but they require clinical follow-up because of their risk of hypertension. Chronic disease measures available for this study included provider documentation of coronary artery disease (CAD) and diabetes (including evidence of glucosuria on urinalysis), and evidence of anemia (by hemoglobin values of <13 g/dL in men and <12 g/dL in women) (23).

In describing the population that used services, we first determined the number of refugees seen in RHAP from 2001 through 2005 by year of US entry and the number of asylees seen by year in which status was granted. We then described all people who completed RHAP screening by sex, visa category, age, and region of origin. The 5 regions of origin represented 92 countries.

For our main analyses, we determined the prevalence of obesity/overweight, hypertension, CAD, diabetes, and anemia, overall and by region of origin. We also determined the prevalence of obesity/overweight by age group. We used SAS version 9.1 (SAS Institute, Inc, Cary, North Carolina) to conduct multivariate logistic regression to examine associations of CAD and diabetes with being from the Europe and Central Asia region (including countries of the former Soviet Union and the former Yugloslavia), adjusting for age, sex, and BMI as covariates in the model. Visa category was not included in regression models because of the low numbers of asylees in the overall population and concerns about covariation of visa category with the more robust place-of-origin variable. Among refugees only, we also examined the proportion of documented diagnoses of CAD and diabetes that originated in reports from overseas medical examinations performed before US arrival and participation in RHAP screening.

## Results

Of the 5,141 adult refugees and asylees with dates of entry from 2001 through 2005, RHAP documentation was available for 4,239 (82.5%) who completed health screening. Those who completed RHAP screening were similar to those who did not with respect to mean age (37.7 vs 36.5 years) and sex (49.8% vs 52.4% women). They differed in respect to country of origin (43.8% of completers vs 25.3% of noncompleters were from Europe and Central Asia) and visa category (11.2% of completers vs 13.5% of noncompleters were asylees).

The distribution of visa categories among people who received RHAP services varied by year of eligibility (Figure) and reflect the US allocations of visas each year (24). The reduced numbers of refugees from 2001 through 2005 reflect the government's limited processing of visa applications of refugees overseas after the September 11 terrorist attacks. The top 2 regions of origin of all people who completed RHAP screening were Europe and Central Asia and Africa (Table 1). Among the 3,765 refugees and the 474 asylees who completed RHAP screening, the top regions of origin respectively were Europe and Central Asia (47.8%) and Africa (52.7%). Compared with asylees, refugees had a higher mean (SD) age (38.8 [16.0] years vs 34.8 [10.5] years for asylees) and a slightly lower proportion of women (50.3% vs 52.7% of asylees).

**Figure:**
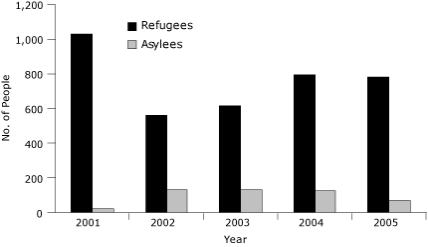
Distribution of refugees and asylees who received health assessment services in Massachusetts, 2001-2005 (N = 4,239).

**Table:** 

**Year**	**Refugees**	**Asylees**	**Total**
2001	1,027	21	1,048
2002	556	131	687
2003	614	131	745
2004	792	123	915
2005	776	68	844
Total	3,765	474	4,239

We found differences in sex, visa category, and mean age by region of origin (Table 1). Women accounted for approximately half of asylees/refugees from all 5 regions. Asylees accounted for as little as 3.1% of people from Europe and Central Asia and as much as 28.5% of those from Latin America and the Caribbean. The mean age was highest for people from Europe and Central Asia and lowest for those from Africa.

Overall, almost one-fifth of this sample was obese, and more than one-fourth was overweight (Table 2). The largest proportions of obese and overweight people were from Europe and Central Asia. Among 18- to 49-year-olds, more than one-fourth were overweight and 15.9% were obese. Among 50- to 79-year-olds, 34.3% were overweight and 31.5% were obese. Almost one-fourth had hypertension diagnoses, again with documentation highest among people from Europe and Central Asia. Those from East and Southeast Asia had the lowest prevalence of obesity/overweight (3.6% and 21%, respectively) and the lowest prevalence of hypertension.

Documented chronic diseases varied by region of origin (Table 2). People from Europe and Central Asia contributed disproportionately to documented diagnoses of CAD. Anemia was highest among people from Africa and lowest among those from East and Southeast Asia.

In logistic regression models adjusting for age, sex, and BMI, people from Europe and Central Asia were significantly more likely than others to have CAD (adjusted odds ratio [AOR], 5.55; 95% confidence interval [CI], 2.95-10.47). Additionally, they were slightly less likely to have diabetes (AOR, 0.74; 95% CI, 0.49-1.13), but this latter finding was not significant.

Among the total of 157 diagnoses of CAD, 153 were among refugees rather than asylees. Most (81%) of these 153 refugee diagnoses had been entered in the RHAP database from overseas medical examination reports rather than from new findings during RHAP screening. Most (95%) of these 153 refugee diagnoses were among people from Europe and Central Asia. Among the total of 131 diagnoses of diabetes, 71 were among refugees. Almost half (49%) of these 71 diagnoses had been entered in the RHAP database from overseas medical examination reports. As with CAD, most (61%) of the 71 refugee diagnoses were among people from Europe and Central Asia.

## Discussion

Region of origin was strongly associated with prevalence of risk factors for and presence of the chronic diseases assessed in this study, with the exception of diabetes. Associations with visa category were less consistent; however, because of their high concentration among people from Europe and Central Asia, refugees were significantly more likely than asylees to have certain risk factors or chronic diseases, particularly CAD. We found that almost one-fifth of our sample were obese, more than one-fourth were overweight, and almost one-fourth had hypertension. In comparison, the overall rates of documented CAD and diabetes were low. Refugees and asylees from the Europe and Central Asia region had the highest prevalence of obesity/overweight and hypertension and were more than 5 times more likely to have documented CAD compared with those from other regions. Regional differences in anemia prevalence in this young study sample were also apparent, suggesting other underlying chronic disease or nutritional deficiencies that varied by region. 

Few studies of chronic disease among United States refugee populations exist, necessitating comparison of our findings with those of studies of immigrants as well as refugees. A recent study of 459 refugee psychiatric patients found the prevalence of hypertension and diabetes to be 42.0% and 15.5%, respectively (25). This was significantly higher than US norms and was especially pronounced in people younger than 65 years. Rates of obesity were also high, especially among Bosnians (54.5%), similar to our findings among people from Europe and Central Asia. In another study of Russian-speaking adult immigrants in New York, 53.8% had hypertension and 33.2% were obese, significantly higher prevalence rates than among other non-Hispanic whites after age adjustment (26). Lastly, in a nationally representative study of 6,421 adult immigrants with newly acquired legal permanent residence, the adjusted prevalence of obesity/overweight ranged from 36.5% to 65.9% for men and from 21.7% to 53.3% for women across all regions (27). The prevalence was lowest among men and women from Asia (similar to our study findings) and highest among men from the Latin America and Caribbean region and women from the Middle East and North Africa region. Higher prevalence of risk factors and chronic diseases found in the studies above may be related to more acculturation to US lifestyle (28,29).

The low prevalence of CAD and diabetes found in our study may be accurate in this young population of primarily recently arrived refugees. It may also indicate inadequate time or resources for diagnosis of disease during either overseas or US health screening. Despite the overall low prevalence of CAD, the significantly increased likelihood of CAD among people from Europe and Central Asia compared with those from other regions may reflect the high burden of this disease in Russia, where cardiovascular disease is the leading cause of death (16). High rates of CAD in Russia may be due in part to high rates of smoking and hypertension in this region. One unexpected finding was the lower (but not significant) likelihood of diabetes in people from Europe and Central Asia compared with all other regions. This could be related to distinct differences in dietary patterns in Europe and Central Asia, including an increase in moderate alcohol consumption, which has been postulated in a meta-analysis of epidemiologic data on diabetes risk factors to reduce risk for development of type 2 diabetes (30).

One of this study's main strengths was the large sample size and demographic diversity of the refugees and asylees in Massachusetts. The large numbers of refugees and asylees in the RHAP database facilitated comparisons of the prevalence of risk factors and diagnoses of chronic diseases across regions of origin that could not have been done using a sample drawn from a single clinic. These comparisons are likely generalizable to other refugees and asylees resettling across the United States during the study period. However, they may be less generalizable to refugee/asylee populations entering the United States in other years because the regions of origin represented, as well as the diet and lifestyle patterns in a given region, may change over time.

The data available from the RHAP database were somewhat limited. Although refugees in the RHAP are typically seen within 90 days of arrival in the United States, it is likely that asylees had been in the United States for a longer time before RHAP screening, thus increasing chances of acculturation to US diet and lifestyle (7). However, data were not available to quantify these times more precisely. In addition, CAD, diabetes, and hypertension may have been underreported because these diagnoses were based on provider documentation either from overseas medical examinations or domestic health screening. On the other hand, we were able to extract more objective measures from the RHAP database to quantify obesity/overweight, elevated blood pressure, glucosuria, and anemia.

In summary, although rates of CAD and diabetes were low, this study found a high prevalence of risk factors for chronic disease such as obesity/overweight and hypertension. Findings suggest that refugees and asylees from Europe and Central Asia fall into a high-risk category. Future populations resettling in the United States should be linked to more resources to address their long-term health care needs and to receive culturally appropriate counseling on risk reduction. Further studies may shed more light on differences in risk among different subpopulations of refugees and asylees, but more programs are needed to help establish primary care after domestic health screening. Primary care will increase the overall health of these populations and the likelihood that they will be able to successfully integrate into United States society over time.

## Figures and Tables

**Table 1:** Most Commonly Represented Countries/Areas Within the 5 Regions of Origin of People Who Received Refugee Health Assessment Services, Massachusetts, 2001-2005 (N = 4,239)

**Countries/Areas**	All, n (% Total)	Women, n (% Region)	Asylees n (% Region)	Age, Mean (SD), y
**Europe and Central Asia**	1,858 (43.8)	980 (52.7)	57 (3.1)	43.8 (17.5)
Former Soviet Union	1,634 (38.5)	871 (53.3)	32 (2.0)	44.8 (17.8)
Former Yugoslavia	195 (4.6)	94 (48.2)	1 (0.5)	35.7 (12.9)
Albania	29 (0.7)	15 (51.7)	24 (82.8)	37.4 (11.5)
**Africa**	1,497 (35.3)	704 (47.0)	250 (16.7)	31.8 (13.1)
Somalia	493 (11.6)	242 (49.1)	19 (3.9)	34.3 (14.8)
Liberia	305 (7.2)	176 (57.7)	22 (7.2)	31.9 (13.6)
Sudan	220 (5.2)	39 (17.7)	7 (3.2)	25.5 (7.7)
**East and Southeast Asia**	338 (8.0)	164 (48.5)	55 (16.3)	36.3 (11.6)
Vietnam	185 (4.4)	91 (49.2)	0 (0.0)	35.6 (10.0)
Cambodia	99 (2.3)	53 (53.5)	49 (49.5)	36.9 (13.4)
Burma	24 (0.6)	5 (20.8)	1 (4.2)	36.3 (10.0)
**Near East and South Asia**	213 (5.0)	107 (50.2)	17 (8.0)	35.0 (12.8)
Afghanistan	135 (3.2)	78 (57.8)	0	36.2 (13.5)
Iran	44 (1.0)	14 (31.8)	7 (15.9)	32.4 (12.4)
Iraq	25 (0.6)	11 (44.0)	3 (12.0)	34.0 (8.0)
**Latin America and Caribbean**	333 (7.9)	154 (46.3)	95 (28.5)	33.2 (9.6)
Haiti	233 (5.5)	97 (41.6)	60 (25.8)	31.8 (7.7)
Cuba	44 (1.0)	19 (43.2)	0	38.9 (12.4)
Colombia	42 (1.0)	30 (71.4)	27 (64.3)	36.6 (11.5)
**All regions**	4,239 (100.0)	2,109 (49.8)	474 (11.2)	37.7 (15.8)

**Table 2:** Medical Conditions by Region of Origin of People Who Received Refugee Health Assessment Services, Massachusetts, 2001-2005 (N = 4,239)^a^

Region	Risk Factors, n (% Region)			Chronic Diseases, n (% Region)		

	Obesity^b^	Overweight^b^	HTN^c^	CAD	Diabetes^d^	Anemia^e^
Europe and Central Asia	508 (27.3)	580 (31.2)	599 (32.2)	145 (7.8)	65 (3.5)	176 (9.5)
Africa	199 (13.3)	362 (24.2)	245 (16.4)	8 (0.5)	37 (2.5)	294 (19.6)
East and Southeast Asia	12 (3.6)	71 (21.0)	33 (9.8)	2 (0.6)	12 (3.6)	18 (5.3)
Near East and South Asia	29 (13.6)	58 (27.2)	25 (11.7)	1 (0.5)	6 (2.8)	26 (12.2)
Latin America and Caribbean	62 (18.6)	104 (31.2)	58 (17.4)	1 (0.3)	11 (3.3)	30 (9.0)
All regions	810 (19.1)	1,175 (27.7)	960 (22.6)	157 (3.7)	131 (3.1)	544 (12.8)

Abbreviations: HTN, hypertension; CAD, coronary artery disease.

a χ^2 ^Statistical testing was used to determine association between having a given chronic disease or risk factor and region of origin: *P* = .51 for diabetes, *P* < .001 for all other conditions.

b Obesity defined as body mass index ≥30 kg/m^2^, overweight defined as 25.0-29.9 kg/m^2^.

c Defined as diagnosis of HTN or measurement of systolic blood pressure ≥140 mm Hg.

d Included presence of glucose on urinalysis.

e Hemoglobin <13 g/dL or hematocrit <41% (men) and hemoglobin <12 g/dL or hematocrit <36% (women).
